# Improving the performance of spray operators through monitoring and evaluation of insecticide concentrations of pirimiphos-methyl during indoor residual spraying for malaria control on Bioko Island

**DOI:** 10.1186/s12936-020-3118-y

**Published:** 2020-01-21

**Authors:** Godwin Fuseini, Hanafy M. Ismail, Michael E. von Fricken, Thomas A. Weppelmann, Jordan Smith, Rhiannon Agnes Ellis Logan, Folasade Oladepo, Kyle J. Walker, Wonder P. Phiri, Mark J. I. Paine, Guillermo A. García

**Affiliations:** 1 Medical Care Development International, Malabo, Equatorial Guinea; 2 Liverpool School of Tropical Medicine, Liverpool, UK; 3 Dept. of Global and Community Health, George Mason University, Fairfax, VA USA; 4 Herbert Wertheim College of Medicine, Florida International University, Miami, FL USA; 5 Medical Care Development International, Silver Spring, MD USA

**Keywords:** Malaria, Indoor residual spraying, Vector control, Quality control, Bioko Island

## Abstract

**Background:**

Quality control of indoor residual spraying (IRS) is necessary to ensure that spray operators (SOs) deposit the correct concentration of insecticide on sprayed structures, while also confirming that spray records are not being falsified.

**Methods:**

Using high-performance liquid chromatography (HPLC), this study conducted quality control of the organophosphate insecticide pirimiphos-methyl (Actellic 300CS), during the 2018 IRS round on Bioko Island, Equatorial Guinea. Approximately 60 SOs sprayed a total of 67,721 structures in 16,653 houses during the round. Houses that were reportedly sprayed were randomly selected for quality control testing. The SOs were monitored twice in 2018, an initial screening in March followed by sharing of results with the IRS management team and identification of SOs to be re-trained, and a second screening in June to monitor the effectiveness of training. Insecticide samples were adhesive-lifted from wooden and cement structures and analysed using HPLC.

**Results:**

The study suggests that with adequate quality control measures and refresher training, suboptimal spraying was curtailed, with a significant increased concentration delivered to the bedroom (difference = 0.36, P < 0.001) and wooden surfaces (difference 0.41, P = 0.001). Additionally, an increase in effective coverage by SOs was observed, improving from 80.7% in March to 94.7% in June after re-training (McNemar’s test; P = 0.03).

**Conclusions:**

The ability to randomly select, locate, and test houses reportedly sprayed within a week via HPLC has led to improvements in the performance of SOs on Bioko Island, enabling the project to better evaluate its own performance.

## Background

Indoor residual spraying (IRS) remains a critical tool in reducing the global burden of malaria in endemic countries [1]. Recent studies in different epidemiological settings have all shown that IRS has substantially reduced infant and child mortality [2–5]. It functions primarily by killing endophagous mosquitoes when they land on sprayed surfaces, as well as by deterring feeding mosquitoes from entering the house completely [6]. An estimated 663 million cases of malaria were prevented between 2000 and 2015, 68% of which are attributable to insecticide-treated nets (ITNs) and 10% to IRS [7]. Despite the success, IRS campaigns have had in reducing the burden of malaria, many factors can affect the efficacy of IRS including insecticide resistance, costs of spraying, and the level of training spray operators (SOs) receive [6, 8]. The World Health Organization (WHO) has recommended IRS as a primary vector control intervention for reducing and interrupting malaria transmission, urging its use in national malaria control strategies in countries where the intervention is appropriate [9]. Maximizing the effect of IRS in reducing malaria transmission however requires efficient delivery of effective insecticide at large-scale [10, 11].

The Bioko Island Malaria Control Project (BIMCP) has committed to reducing the burden of malaria on Bioko Island through methods such as concerted vector control, improved case management, and various educational interventions for the past 15 years. As part of the Equatorial Guinea insecticide resistance management plan, the BIMCP monitors the resistance profile of the malaria vector on Bioko Island. Recent studies indicate that vectors remain susceptible to organophosphates and carbamates insecticides, but are resistant to pyrethroids and organochlorines [12]. In addition, samples of the IRS insecticide are sent to a reference laboratory for quality control, to verify if the formulation meets the requirements as specified by WHO. IRS coverage at the community level is estimated at around 80% for Bioko Island [13]. As a result of the vector control interventions and case management since 2004, *Plasmodium falciparum* prevalence in the 2 to 14-year-old age group has dropped from 45% to 12.5% in 2016 in 18 sentinel sites and 10.9% for the whole island [14–16]. Actellic 300CS, a microencapsulated organophosphate insecticide with pirimiphos-methyl as the active ingredient, was introduced for IRS on Bioko Island in 2017 after a study conducted in 2015 revealed that the Bioko Island malaria vectors had developed both target site and metabolic resistance to pyrethroids [17]. The residual effectiveness of Actellic 300CS under controlled spraying on Bioko Island was determined to be at least 8 months [13]. IRS with Actellic 300CS has been shown to reduce malaria transmission in recent studies [5], when applied correctly at the recommended dose of 1·0 g/m^2^ on sprayed structures [18]. Preserving these insecticides from vector resistance requires constant monitoring and efficient delivery during IRS, making quality control and training critical components of successful programmes. This study focuses on using high-performance liquid chromatography (HPLC) to monitor organophosphate concentrations and assess intra-operational target dosage of SOs during the 2018 IRS round on Bioko Island. The findings from this study will be helpful to illustrate the benefits of quality monitoring and periodic re-training of spray operators during IRS operations, aiding in the elimination of malaria on Bioko Island.

## Methods

### Study site

The Bioko Island Malaria Control Project (BIMCP) has been implemented since 2004 by the non-profit organization Medical Care Development International (MCDI) on the Island of Bioko of the Republic of Equatorial Guinea, where malaria transmission occurs throughout the year. Since 2004, IRS and LLINs have been used as the main vector control interventions. Between 2004 and 2014, Island-wide IRS was conducted using either pyrethroids or carbamates insecticides. Since 2015, a stratified approach has been used with IRS focusing on areas with high parasite prevalence.

### Spraying operations

In 2018, the BIMCP IRS programme deployed a total of 60 spray operators who worked in previous spray rounds. All SOs were trained immediately before the start of the 25th spray round on Bioko in February 2018. A total of 67,721 structures in 16,653 houses were sprayed over a five-month period, with structures defined as at least three walls and a roof. This definition includes rooms of a house, terraces, and adjacent structures to the house. Roughly 90% of IRS was carried out in Malabo, the capital city, where 90% of the population resides. All underperforming SOs, based on results from the first round of HPLC quality control, received a refresher training that was identical to the original, first training, and was based on standard WHO practices, which had to be completed before being allowed to return to the field, with the added warning that continued poor performance may result in suspension or job termination. Quality control monitoring for this study was conducted for both periods of observation using HPLC in 2018.

### Sampling method

Data on sprayed structures were recorded on sprayer reporting cards, using the BIMCP mapping system to record the household’s unique identifier [19]. The sprayer card information was then entered into the Campaign Information Management System (CIMS), an Android-based application built around the household database and used as a tool for identifying and locating households targeted for interventions and surveying. Houses that SOs reported spraying were then randomly selected within one-week post spraying. A sampling of the insecticide from the surface of the walls was carried out in living rooms and bedrooms that were reported as sprayed. For each period of monitoring, three samples were randomly taken from the sprayed walls in each room using adhesive strips with four glue dots on each strip: one strip at the top of the sprayed wall, one in the middle, and the other at the bottom. Thus, for each spray operator, six samples were taken from the living room and bedroom of each house. Samples included both cement (n = 390) and wood (n = 324) surfaces and were collected after each spray period in March and June with identified underperforming SOs re-trained in between operations. All the samples were stored at 4 °C and sent to Liverpool School of Tropical Medicine (LSTM) for HPLC analysis. The surfaces were sprayed by 60 spray operators; 57 sprayed two households each (12 Samples per SO), two sprayed one household each (six samples per SO), and one sprayed three households (18 samples). All testing occurred within a single spray campaign (Feb–July 2018), with the first HPLC screen occurring in March, roughly 20% into the campaign, and the second HPLC screen occurring in June, roughly 70% into the campaign.

### HPLC analysis

The strip of four glue dots were stuck to filter paper (Whatman no 1) to avoid self-folding during storage and shipping. The four glue dots were individually cut out using a hole-punch (radius 0.365 cm^2^), giving a total filter/glue dot area of 4.6 cm^2^ and transferred to a 10 ml glass tube. Primiphos-methyl was extracted from the glue dots by the addition of 5 ml acetone containing 100 µg/ml dicyclohexyl phthalate (DCP) (Sigma Aldrich, UK) as an internal standard. The glass tubes were sealed with tin foil followed by capping with lid and sonicated for 15 min at room temperature. 1 ml of the insecticide extract was transferred to a clean glass tube and evaporated to dryness under compressed air at 60 ℃. Samples were re-suspended in 1 ml acetonitrile and vortexed for 1 min, transferred to 1·5 ml Eppendorf tubes and centrifuged at 13,000 rpm for 20 min at room temperature. 100 µl of the supernatant was transferred to a Chromacol 300 µl glass vial (Thermo Scientific, UK). High-performance liquid chromatography (HPLC) analysis was performed by injection of 20-μL aliquots of the extract on a reverse-phase Hypersil GOLD C18 column (75 Å, 250 × 4.6 mm, 5-μm particle size; Thermo Scientific, UK). To separate primiphos-methyl and DCP a mobile phase of acetonitrile/water (70/30 v/v) was used at a flow rate of 1 mL.min^−1^. Peaks were detected at 232 nm with the Ultimate 3000 UV detector (Dionex) and analysed with Dionex Chromeleon software. The quantities of primiphos-methyl were calculated from standard curves established with known concentrations of primiphos-methyl authenticated standards (PESTANAL^®^, analytical standard, Sigma-Aldrich, UK) and corrected against internal standard (DCP) readings. Final insecticide content in g/m^2^ was corrected using a 15% surface active ingredient extraction efficiency estimation. This was calculated by application of Actellic 300CS to the rough side of tiles that were used as a laboratory reference surface for estimating sampling efficiency. The extraction efficiency of 15% was based on primiphos-methyl recovery with glue dots on rough tiles dosed with Actellic 300CS in the range 0.3–3 g/m^2^. The average recovery across the range was 16.5 ± 5.7% (Additional file 1: Table 1). This was rounded down to 15% to take account of the lower extraction efficiency (12%) in the 1 g/m^2^ target range.

### Statistical analyses

Statistical comparison of the residual concentration on surfaces between different rooms (bedroom or living room), wall heights (upper, middle, or lower), or surface composition (concrete or wood) was conducted with linear regression models with and without adjustment via the inclusion of all covariates into a single model. The surfaces were categorized as sub-optimal (concentration less than 0.5 g/m^2^), acceptable (concentration > 0.5 g/m^2^ and < 1.5 g/m^2^), and unnecessary (concentration more than 1.5 g/m^2^) and compared between March and June operations before and after refresher training of operators via contingency tables (using Fischer’s exact test). Similarly, the concentration of residuals was averaged by household to determine how many households received adequate coverage or excessive coverage between operations. Finally, pairwise comparisons of individual operators before and after retraining were evaluated graphically and with McNemar’s test for matched pairs. Only the 57 SOs that sprayed the same amount of treatments before and after were considered in this sub-analysis. An alpha level of 0.05 was used to denote statistical significance and all analyses were conducted using STATA (Stata Corp, College Station, TX, USA).

## Results

**Fig. 1:**
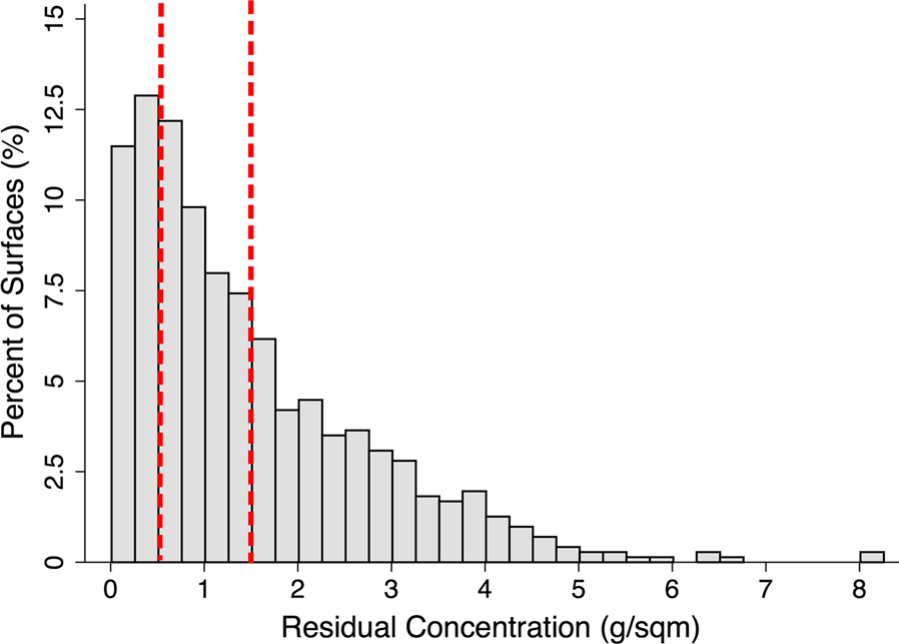
Distribution of IRS pesticide concentrations from household surfaces. Histogram of residual concentration of pesticide sampled from 119 households after organized malaria elimination efforts. Concentrations sampled from surfaces (n = 714) were determined by HPLC and presented with reference lines for samples below 0.5 g/m^2^ (sup-optimal dose) and above 1.5 g/m^2^ (no additional benefit)

**Fig. 2:**
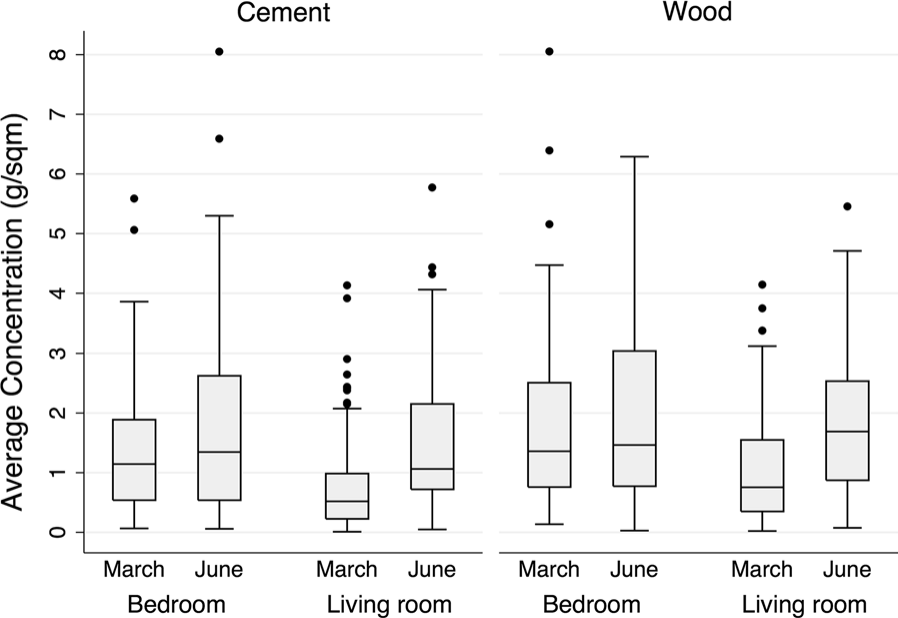
Comparison of residual concentrations by room and surface between operations. The average concentrations of pesticide residuals are presented in a box and whisker plot by room (bedroom or living room) and operation periods (1 for March and 2 for June) by surface type (concrete or wood)

Approximately 1 week after each IRS period was completed, samples (n = 714) were collected from households (n = 119) and sent for analyses via HPLC. The distribution of IRS concentrations is presented in Fig. 1, along with categorical summaries by room, location, surface, and operational period in Table 1 and a box plot in Fig. 2. There was a higher concentration of pesticide residual delivered to the bedroom than the living room (Difference = 0.4 g/sqm^2^, P < 0.001) in March, which was no longer observed after retraining in June (P = 0.30). Additionally, there was a higher concentration of pesticide residual delivered to wood surfaces than concrete (Difference = 0.4 g/sqm^2^ P < 0.001) in March, which was also followed by a non-significant difference after retraining in June (P = 0.19). No differences were observed between wall section (top, middle, or bottom) of the surface sprayed in total nor in either period analysed separately (P > 0.1). When the room, wall section, or surface type were included into a multivariate regression model stratified by March or June IRS operation; there was a significant increased concentration delivered to the bedroom (Difference = 0.36, P < 0.001) and wood surfaces (Difference 0.41, P = 0.001), but not by wall section (P > 0.1). Upon retraining in June, there were no significant differences in the concentrations by room (P = 0.30), surface type (P = 0.187), or sample location within surface (P > 0.35).

**Table 1:** Summary of concentrations by variables

Variables	n	Mean	Median	STDDEV
Room				
Bedroom	357	1.696	1.328	1.387
Living room	357	1.296	0.927	1.131
Location				
Top	238	1.548	1.187	1.321
Middle	238	1.543	1.186	1.269
Bottom	238	1.398	0.971	1.248
Surface				
Cement	390	1.313	0.928	1.205
Wood	324	1.717	1.378	1.333
Spray period				
March	360	1.207	0.839	1.146
June	354	1.791	1.457	1.342
Total	714	1.497	1.108	1.280

The concentration of residual determined by HPLC is presented with the number of samples included, the mean, median, and standard deviation for the type of room, location on surface, surface type, and for the two observed periods of spraying in March and June 2018

**Fig. 3:**
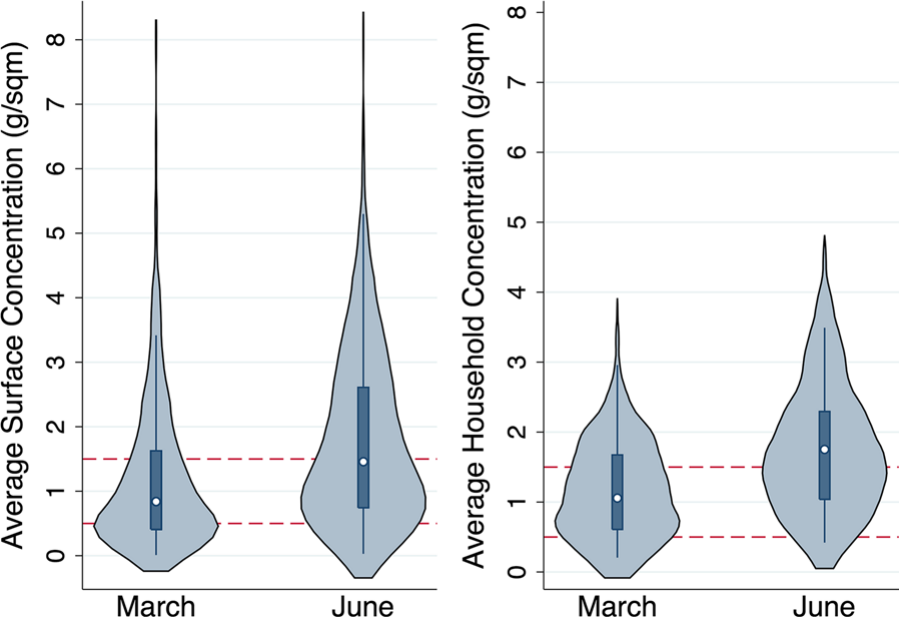
Comparison of surface residual and average household concentrations concentration between March and June. The average household concentration from six surfaces is presented for the March and June IRS operations with respect to the recommended dose of 0.5 g/m^2^ and a dose with no added insecticidal benefit of 1.5 g/m^2^. Those within the appropriate range are shown in grey, those either too high or low are shown in red. The average surface concentration applied by spray operators is presented for the March and June IRS operations with respect to the recommended dose of 0.5 g/m^2^ and a dose with no added insecticidal benefit of 1.5 g/m^2^. Those within the appropriate range are shown in grey, those either too high or low are shown in blue and red, respectively

The average residual concentration of pesticides deposited on household surfaces between March and June operations is presented in Fig. 3. In the first period of spraying in March 2018, 32.2% of surfaces (116/360) had less than the recommended 0.5 g/m^2^. Following a re-training, the second period of spraying in June 2018 had only 15.3% of surfaces (54/354) with less than the recommended dose. This corresponded to an estimated 62% reduction in the number of surfaces with sub-optimal dosing (OR = 0.38; 95% CI 0.26 to 0.55), which was statistically significant (P < 0.001). After re-training, the samples collected showed a significant increase (P < 0.001) in surfaces with more than the necessary dose of 1.5 g/m^2^ from 28.3% (102/360) to 48.3% (171/354); an estimated 2.4-fold increase (OR = 2.36; 95% CI 1.73 to 3.22).

The average residual concentration of pesticides deposited into each house between March and June operations is presented in Fig. 3. In the first period of spraying in March 2018, 18.3% of households (11/60) had less than the recommended 0.5 g/m^2^. Following a re-training, the second period of spraying in June 2018, only 5.1% of households (3/59) had less than the recommended dose. This corresponded to estimated 76.2% reduction in the number of houses with sub-optimal dosing (OR = 0.24; 95% CI 0.06 to 0.90), which was statistically significant (P = 0.035). This corresponded to a significant increase (P = 0.036) in houses with more than the necessary dose of 1.5 g/m^2^ from 36.6% (22/60) to 55.9% (33/59); an estimated two-fold increase (OR = 2.19; 95% CI 1.05 to 4.57).

**Fig. 4:**
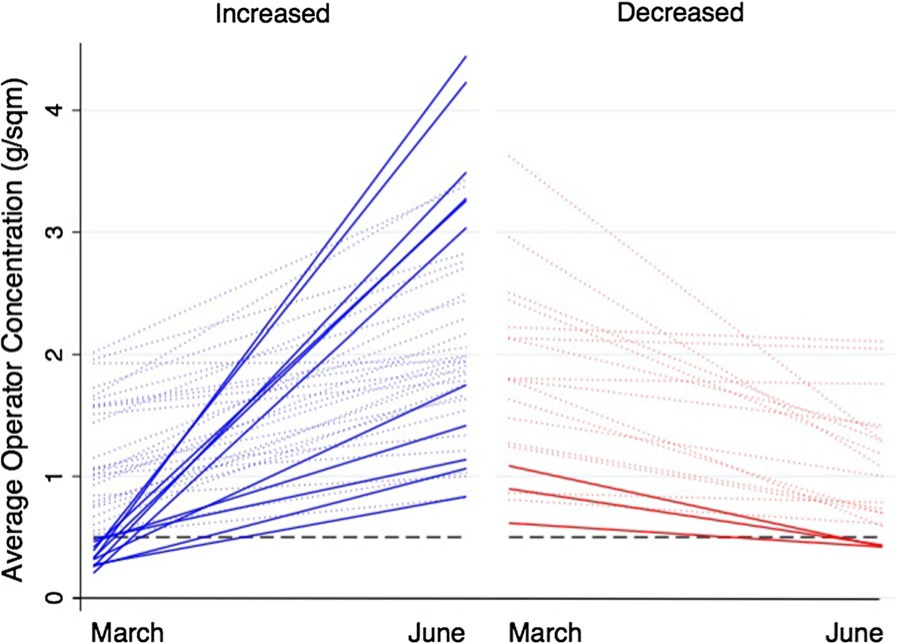
Comparison of IRS concentrations between March and June by operator. The average residual pesticide concentration from spray operators is presented for the March and June IRS operations. The solid lines represent operators that were either below the recommended level and improved performance to meet the 0.5 g/m^2^ dose (left, blue), or those that met the 0.5 g/m^2^ dose and decreased performance to below the 0.5 g/m^2^ dose (right, red). Dotted lines represent operators either increased (left, blue), or decreased concentration (right, red) between operations that maintained the minimum dose of 0.5 g/m^2^ on both operations

The change in the performance of each operator before and after re-training is presented in Fig. 4. Upon comparison of the differences in concentrations deposited by spray operators before and after the March and June IRS operations, 75.4% (43/57) dispensed an appropriate dose in both periods with no operators dispensing an inadequate dose in both March and June. Among the discordant pairs, 100% (11/11) of operators that dispensed a sub-optimal dose in March sprayed an appropriate dose in June; 6.5% (3/46) of operators that previously dispensed an adequate dose in March dispensed an inadequate dose in June. This corresponded to an increase in effective coverage by spray operators from 80.7% of operations in March to 94.7% in June after re-training (McNemar’s test; P = 0.03).

## Discussion

This study found that with adequate quality control and refresher training, suboptimal spraying was curtailed, with a significant increase in concentration delivered to the bedroom (difference = 0·36, P < 0·001) and wooden surfaces (difference 0.41, P = 0.001). Additionally, an increase in effective coverage by spray operators was observed, improving from 80.7% in March to 94.7% in June after re-training (McNemar’s test; P = 0.03). Spraying houses is a complex process that requires appropriate training and strict adherence to procedures. Challenges in IRS quality control have traditionally resulted from SOs depositing sub-optimal concentration of insecticide on sprayed structures, which can result in vectors developing resistance to the insecticide; or outright falsification of spray records leaving no residual insecticide for household protection [20–24]. Training SOs and supervisors on proper techniques and best practices is critical to the success and overall impact of IRS programmes.

The mass effect of IRS in protecting communities is realized when coverage rates exceed 85% in at-risk populations [25]. However, even with good training and adequate supervision, it can be difficult to monitor at scale whether the correct dosage of insecticide has been deposited during IRS campaigns. Through randomization and using HPLC, this study was able to conduct quality control that had a direct impact on sprayer performance.

Several studies have shown that spray operators missed the target dose of insecticide deposits during and post IRS [21, 26, 27]. Factors that influence the residual effectiveness of insecticide on sprayed surfaces include vector susceptibility to insecticide, types of surfaces sprayed, amount of insecticide applied, formulation of the insecticide, and the weather condition of the location [28–30]. According to the 2017 BIMCP Malaria Indicator Survey (MIS), 51.8% of the buildings on the Island are constructed with cement and 47.1% with wood. During this study, higher concentrations of insecticide were recovered from wood than cement surfaces, with no difference by wall height. Other studies have found that wood surfaces have longer residual effect than cement white wash surfaces after 180 days of post-application [10, 26]. This study speculates that the SOs tend to increase the rate of spaying on non-absorbent surfaces such as painted walls. The differences in the concentrations between bedrooms and living rooms were not explained by surface composition, but could potentially be due to sprayers consistently applying more protection in the bedroom due to ease of applying spray in areas with less furniture. Regardless, after retraining, these differences were no longer observed.

All the houses monitored during this study had insecticide deposits, indicating SOs reports of spraying were not falsified. Although the current IRS coverage on Bioko Island remains relatively high in both rural Bioko (92%) and urban Malabo (80%), depositing lower concentrations of insecticide during spraying could affect the long-term protective effect of IRS on the population. This study indicates that quality control of IRS, paired with refresher training of under-performing SOs, are critical additions to IRS programmes and should be considered in similar settings elsewhere in Africa. Of concern, a proportion of SOs that were under spraying in period one, overcompensated in period two after refresher training, depositing average amounts as high as 4.4 g/m^2^, which is well above the recommended concentration. The SOs overcompensation could be related to their perspective that they do not wish to be recalled for training due to sub-optimal spraying, and the rest of the team is aware of this group, but this warrants further investigation.

This method of quality control is best suited for IRS programmes that are spread over several months, since it can take up to 3 weeks to collect, ship, and analyse samples via HPLC, which can cause a slight delay in immediate corrective action. Additionally, HPLC costs came to roughly $60–70 per sample, with 12 samples collected per sprayer (~ $720–840 per sprayer), which may be out of reach for underfunded programmes with hundreds of sprayers. However, no significant difference in concentration was observed based on testing of different sections of the wall (top, middle, and bottom); thus, the number of samples taken per sprayer could be reduced, lowering overall costs. Additionally, this study was unable to determine if sprayer performance improved due to the additional training, or due to SOs being aware of the quality control tests taking place. It is possible that SO performance increased across the board because SOs were made aware that random concentration testing was occurring, resulting in an increase in diligence not because of being better trained, but from a phenomenon known as the Hawthorne effect [31]; a change in behaviour by the subjects of a study due to their awareness of being observed, or in this case an improvement in spraying since SOs were aware they were being observed and monitored.

Lastly, the insecticide recovery is surface and concentration dependent. Since an estimated 15% recovery based on a proxy surface was used, caution must be taken into consideration in interpreting the accuracy of the amounts recovered from field surfaces. The differences between wood and cement may be linked to different surface extraction efficiencies. Thus, the accuracy may be improved in the future by measuring the extraction efficiencies from individual field surfaces.

## Conclusion

Quality control and refresher training resulted in a dramatic improvement in sprayed concentration and sprayer effectiveness. This study found that with adequate quality control and refresher training, suboptimal spraying was significantly reduced, with an increase in effective coverage by spray operators from 80.7% in March to 94.7% in June post re-training (McNemar’s test; P = 0.03). This study shows that while using HPLC is expensive, the benefits of monitoring SOs to reduce the likelihood of falsified spray data coupled with the ability to provide corrective action to SOs depositing suboptimal amounts, should motivate other programmes to include additional IRS quality control.

## Supplementary information

**Additional file 1: Table 1.** Comparison of primiphos-methyl recovery by Bostik adhesive glue dots from rough tile surface treated with various concentrations (0.25–3 g/m^2^) of Actellic 300CS^®^ formulation.

## Data Availability

The datasets used and/or analysed during the current study are available from the corresponding author on reasonable request.

## Abbreviations

IRS: indoor residual spraying
SOs: spray operators
HPLC: high performance liquid chromatography
ITNs: insecticide-treated nets
BIMCP: Bioko Island Malaria Control Project
MCDI: Medical Care Development International
CIMS: Campaign Information Management System
LSTM: Liverpool School of Tropical Medicine
DCP: dicyclohexyl phthalate
MIS: Malaria Indicator Survey
