# Supplementary material for: Improving the performance of spray operators through monitoring and evaluation of insecticide concentrations of pirimiphos-methyl during indoor residual spraying for malaria control on Bioko Island
Source: Malar J. 2020 Jan 21;19:35. doi: 10.1186/s12936-020-3118-y (PMC6975046; doi:10.1186/s12936-020-3118-y)
Supplement: Supplementary file 1 — Additional file 1: Table 1. Comparison of primiphos-methyl recovery by Bostik adhesive glue dots from rough tile surface treated with various concentrations (0.25–3 g/m2) of Actellic 300CS® formulation. [file 12936_2020_3118_MOESM1_ESM.docx]

**Additional file 1**

**Supplementary Table S1** Comparison of primiphos-methyl recovery by Bostik adhesive glue dots from rough tile surface treated with various concentrations (0.25 – 3 g/m^2^) of Actellic 300CS^®^ formulation.

| **Application rate (g/m2)** | **Extraction Efficiency %^a^** | **Average** | **STDEV** |
| --- | --- | --- | --- |
| 3 | 22.9 | 16.53 | 5.7 |
| 2 | 19.73 |  |  |
| 1 | 12.35 |  |  |
| 0.5 | 11.14 |  |  |

^a^ Average of four replicates
